# Understanding Frailty, Functional Health and Disability among Older Persons in India: A Decomposition Analysis of Gender and Place of Resident

**DOI:** 10.34172/jrhs.2020.20

**Published:** 2020-07-28

**Authors:** Ankit Anand, TS Syamala, Md Illias Kanchan Sk, Navneet Bhatt

**Affiliations:** ^1^Population Research Centre, Institute for Social and Economic Change, Bangalore, India; ^2^Department of Population Policies and Programs, International Institute for Population Sciences, Mumbai, India .; ^3^Indian Institute of Forest Management, Bhopal, India

**Keywords:** Gender, Frailty, Disability, Health

## Abstract

**Background:** We estimated and compared the differences in frailty, disability, and functional limitation among men and women, and among urban and rural dwellers. Further, this study also provides the analysis of key factors influencing frailty, functional limitation and disability among older persons in India.

**Study design:** Two cross-sectional surveys.

**Methods:** WHO-SAGE (2007-10) and BKPAI-2011 (Building Knowledgebase for Population Ageing in India) (2007-10) were used. Oaxaca decomposition method was used to decompose the gender and place of resident differentials. Statistical software RStudio (Version 1.2.1335) was used to perform these analyses

**Results:** The decomposition model was able to explain 46.5%, 41.6% and 46.4% of the difference between frailty, functional limitation and disability among older persons respectively. The key factors, which significantly (*P* <0.05) explained the gap for both frailty and functional limitation, were Education (0.009 &1.24), working status (0.018 & 1.93), physical activity (0.001 & 0.15) and migration (0.018 & 1.98). Higher educational attainment (0.008 & 1.10) and wealth quintile (0.009 & 1.18) in urban areas might be a factors resulting in the lowering of frailty and functional limitations.

**Conclusion:** The poorer functional health among older women can largely be explained by gender differentials in socioeconomic status and consequent empowerment (such as less control of their mobility and financial independence). This implies that efforts to improve gender disadvantages in earlier life stages might get reflected in better health for females in older age.

## Introduction

Women live longer with higher rates of functional limitation and disability compared to men ^1,2^. They have also reported more utilization of health services compared to men in higher-income countries ^3,4^. The aging in low-middle income countries like India has just started and the above 60 yr of age population is estimated to double by 2030 ^5^. Further, India has been going through the rapid urbanization, moving from an agricultural-based economy to industrial and technological driven market while there are health disparities which exits between urban and rural areas ^3,6^. Less physical activity, changing family and social systems present a challenge in realising the functional health problems of older persons. Hence, it is imperative to observe these disparities among older persons in the Indian context. The existing research regarding gender differences in functional health and disability in India is limited. The gender gaps in health may be reduced after controlling for social and demographic factors ^7,8^. Other studies point towards the need for further validation.

In this study, we tried to perform a comprehensive analysis to understand the possible reasons for gender and geography-based disparities in India. This also included an assessment of other factors such as social-demographic, chronic-disease and other risk factors.

The discrimination against women has been present in various forms across the majority part of the world, including in India. Lack of access to basic education and health along with hurdles in getting equal employment opportunities for women has been well documented ^9^. Preference for a male child in addition to the dowry system has been one of the cultural characteristics of the Indian family system ^10^. It has been contributed towards the poor health of women in India, something bolstered by the fact that half of the Indian women are almost lifetime anaemic^11^. The cumulative effect of years of neglect and discrimination may result in complications at older ages, resulting in poor health among older women compared to men ^9^. Although many social and economic transitions such as rising income levels, increasing migration towards urban centers, changing roles for women in education and employment, and shifts from extended family structures to smaller nuclear family units are currently underway in India ^6^, positionality in urban and rural areas also present various hindrances in improving the functional health. Poor environmental conditions, cramped spaces, congested transport and lifestyle-related factors are prevalent in Indian cities, towns and suburban areas^12^. Lack of adequate quality health care, poor economic conditions, unavailability of support and care due to migration are some additional issues for older persons in rural areas ^13^.

Disability and functional health is a major public health challenge for aging populations ^14^. As the proportion of older persons is increasing with the demographic transition underway, it will be important to assess the gender and place of resident differentials in frailty, functional limitation and disability among older persons in India.

The objective of this study was to estimate and compare differences in frailty, functional limitation and disability in men and women, and urban and rural dwellers. The study provides the analysis of important factors influencing frailty, functional limitation and disability among older persons in India.

## Methods

### Study design and size

This analysis is based on cross-sectional data from WHO Study on Global AGEing and Adult Health (SAGE) Wave 1 (2007-10). The analysis was conducted in two phases. The first phase entails preparatory phase, which included a literature review of existing studies in related domains, while the second phase essentially covered the execution of the study based on the framework developed and indexes conceptualized in phase 1.

### Data source

WHO-SAGE (2007-10) and BKPAI-2011 are a nationally representative multi-country (China, Ghana, India, Mexico, Russian Federation and South Africa) study to assess the health and well-being of older persons. In India, respondents were selected from six states—Assam, Karnataka, Maharashtra, Rajasthan, Uttar Pradesh and West Bengal. A multistage, stratified, random sampling design was used. More about the sampling process and SAGE India survey can be obtained from the official report^15^. SAGE collected data from adults' age above 18 years. We have considered the adults age 50 yr and above as older persons. The sample size was individual aged 50 yr and above resulting in 7171 individuals. This paper also utilized data from the United Nations Population Fund survey named "Building Knowledge base of Population Ageing in India (BKPAI)." The survey was conducted in Himachal Pradesh, Kerala, Maharashtra, Odisha, Punjab, Tamil Nadu and West Bengal in the year 2011. The primary sampling units (PSUs) in the rural areas were villages, whereas the urban wards were the PSUs in the urban areas. A list of households with at least one elderly person was prepared, and the prescribed number of elderly households (16 households) was selected through systematic random sampling ^16^. This had resulted in a sample size of 9852 people aged 60 and above. SAGE dataset used age 50 and above as older person population and BKPAI consider aged 60 and above as older person population. Using both datasets and different age cutoffs may provide more validity to the results.

### Construction of frailty Index

As per the criteria in the available literature, around 40 variables were used to create the frailty index. The construction procedure and validation of the frailty index have been explained in some literature ^17-19^. The validation of the index using SAGE data has also been explored and found to be an important indicator for healthy aging ^17,20^. The selected variables can be divided into the following broad categories.

1. Self-rated health: Measure in a scale of 5 (Very good, Good, Moderate, Bad and Very bad)
2. Morbidity: Self-reported morbidity is used for 9 medically diagnosed conditions (Angina, Arthritis, Asthma, Cataract, Chronic Obstructive Pulmonary Disease, Diabetes, Depression, Hypertension and Stroke)
3. Medical symptom: Three variables related to self-reported symptoms in last 30 days were used.
4. Functional Limitation in performing Activities of Daily Living (ADLs) and Instrumental Activities of Daily Living (IADLs); Total 23 variables exhibiting limitation in performing ADLs and IADLs were used.
5. BMI: Underweight (BMI<=18.5) and obesity (BMI>=30.0) were considered as frailty.
6. Week grip strength: Grip strength stratified by sex and BMI was used
7. Timed walk: Slow gait speed defined by less than 0.4 m/sec.

The included variables accommodate different types of variables; such as dichotomous (simple yes/no), ordinal and continuous variables. The ordinal and continuous variables were converted as a certain proportion of the deficit. For example, self-rated health (Very good= 0 Good=0.25, moderate=0.5, bad=0.75, very bad=1.00). For each individual/respondents, these deficits were summed up. The index consists of the sum of these deficits divided by total possible deficit to create frailty index. The construction of frailty index for SAGE data is also explained elsewhere^17,20^.

### Functional limitation score

The measurement of physical disability as an indication of the impact of the disease is commonly seen in research. Measurements of decline in functional status was based on a validated questionnaire about the degree of difficulty with functional activities such as climbing stairs, dressing oneself, rising from a chair, cutting toenails, walking outside and using own or public transport, etc. These functional activities are known as activities of daily living (ADLs). Any limitation in activities of daily living is considered as a functional limitation. ADL difficulties have also been expressed as disability progression. A total of 24 variables were used to construct functional limitation index as per WHO DAS score system ^21^.

### Disability

Self-reported disability was collected in Building Knowledge base of Population Ageing in India (BKPAI) survey. Persons who were either fully/partial difficulties for vision, hearing, speaking and walking were categorized as disabled. It was used as an outcome indicator in the analysis.

### Dependent variable

Other covariates such as age, gender, caste, marital status, religion, education, working status and wealth index were used. Migration was defined as person who is not living at their birthplace. Physical activity, number of chronic diseases and body mass index were calculated for each individual. Tobacco and alcohol use were also utilized as covariates.

### Statistical methods

Univariate and bivariate analysis (Frequencies, percentage, mean and SD) were done to observe the socioeconomic profile, frailty, functional health and disability among older persons. Oaxaca decomposition analysis was used to decompose the gender and place of resident differentials ^22^. Multilevel random effect logistic and linear regression models were performed to know the important determinants of frailty, functional health and disability. The two levels, states and Primary Sampling Units (PSU) were used to adjust for the multilevel structure of the data. Missing data were excluded from the analysis. Statistical software RStudio (ver. 1.2.1335) was deployed to perform these analyses ^23^.

## Results

The mean frailty score, mean functional limitation score and disability prevalence are given in appendix 1. Gender differences can be observed for these indicators. Females have a significantly higher average frailty and functional limitation score when compared to males. The disability rate was also significantly high among women. Urban and rural differences in frailty and functional limitation scores were also significant. However, urban and rural difference for disability was not significant.

Factors responsible for gaps between males and females were ascertained by using Oaxaca Decomposition analysis. Our model was able to explain 46.5% and 41.6% of the gender differences in frailty and functional limitation among older persons respectively (Table 1). Education was positively related to increasing the gap, which suggests that the higher educational attainment among males compared to females was an important factor influencing their health. Further, working status and physical activity were also positively related to the gap in our model for both frailty and functional limitations. The low physical activity and non-working status among females get reflected in their higher frailty and functional limitation scores. Tobacco use was also found to be reducing the gap (negative association) suggesting a higher prevalence of tobacco use among men might increase their frailty and functional limitations. For unexplained differences in frailty and functional health, marital status and working status were found to be significant. State differences in frailty and functional health were also significant with unexplained gender differences.

**Table 1:** Decomposition of frailty and functional health by gender among older persons

**Variables**	**Frailty (n=5347)**		**Functional limitation (n=5390)**	
	**Difference (95% CI)**	***P*** **value**	**Difference (95% CI)**	***P*** **value**
Female	0.228 (0.223, 0.233)	0.000	47.850 (47.221, 48.478)	0.000
Male	0.185 (0.180, 0.189)	0.000	41.363 (40.790, 41.935)	0.000
Difference	0.043 (0.037, 0.050)	0.000	6.487 (5.636, 7.338)	0.000
Explained	0.020 (0.012, 0.028)	0.000	2.700 (1.728, 3.672)	0.000
Unexplained	0.023 (0.013, 0.033)	0.000	3.787 (2.518, 5.056)	0.000
**Explained part**				
Age Group	-0.004 (-0.005, -0.002)	0.000	-0.409 (-0.593, -0.225)	0.000
Place of resident	0.000 (-0.001, 0.000)	0.040	-0.084 (-0.147, -0.022)	0.008
Marital Status	0.002 (0.000, 0.004)	0.042	0.245 (-0.022, 0.513)	0.072
Caste	0.000 (0.000, 0.000)	0.471	0.004 (-0.011, 0.018)	0.613
Religion	0.000 (0.000, 0.000)	0.230	-0.009 (-0.028, 0.011)	0.397
Education	0.009 (0.006, 0.011)	0.000	1.240 (0.930, 1.549)	0.000
Wealth quintile	0.000 (-0.001, 0.001)	0.890	0.015 (-0.075, 0.105)	0.741
Currently working	0.018 (0.015, 0.021)	0.000	1.928 (1.538, 2.318)	0.000
Migration	-0.006 (-0.001, -0.002)	0.006	-0.627 (-1.146, -0.107)	0.018
State	0.000 (0.000, 0.000)	0.272	-0.002 (-0.014, 0.009)	0.701
Physical activity	0.001 (0.000, 0.002)	0.005	0.148 (0.047, 0.249)	0.004
Tobacco use	-0.003 (-0.006, 0.000)	0.034	-0.299 (-0.658, 0.061)	0.104
Alcohol use	0.000 (-0.002, 0.002)	0.717	0.070 (-0.190, 0.331)	0.596
Community engagement	0.003 (0.001, 0.004)	0.000	0.274 (0.089, 0.459)	0.004
Personal engagement	0.001 (-0.002, 0.003)	0.533	0.185 (-0.145, 0.514)	0.271
Body mass index	Not applicable	Not applicable	0.020 (-0.061, 0.100)	0.634
**Unexplained part**				
Age Group	0.002 (-0.014, 0.018)	0.818	1.222 (-0.817, 3.261)	0.240
Place of resident	0.003 (-0.017, 0.022)	0.786	0.605 (-1.863, 3.073)	0.631
Marital Status	0.033 (0.013, 0.054)	0.002	4.140 (1.533, 6.746)	0.002
Caste	0.002 (-0.002, 0.005)	0.318	0.291 (-0.161, 0.743)	0.207
Religion	-0.051 (-0.085, -0.018)	0.003	-7.341 (-11.570, -3.111)	0.001
Education	0.002 (-0.003, 0.006)	0.407	0.122 (-0.423, 0.667)	0.661
Wealth quintile	0.019 (0.003, 0.036)	0.019	1.305 (-0.802, 3.412)	0.225
Currently working	0.030 (0.009, 0.050)	0.004	4.330 (1.725, 6.936)	0.001
Migration	-0.020 (-0.043, 0.002)	0.072	-1.961 (-4.748, 0.827)	0.168
State	0.032 (0.015, 0.049)	0.000	4.233 (2.089, 6.377)	0.000
Physical activity	-0.010 (-0.027, 0.007)	0.239	-1.305 (-3.466, 0.855)	0.236
Tobacco use	0.032 (0.017, 0.046)	0.000	3.427 (1.581, 5.272)	0.000
Alcohol use	-0.001 (-0.023, 0.021)	0.938	0.487 (-2.595, 3.569)	0.757
Community engagement	0.021 (0.000, 0.041)	0.047	3.712 (1.110, 6.315)	0.005
Personal engagement	0.003 (-0.015, 0.021)	0.752	0.684 (-1.547, 2.916)	0.548
Body mass index	Not applicable	Not applicable	0.747 (-3.349, 4.843)	0.721

Further, the decomposition analysis was also done to examine the gaps among rural-urban inhabitants. Our model explains 37.5% and 38.3% of the difference between urban and rural areas among older persons in frailty and functional limitation respectively (Table 2). Wealth index and education were positively influencing the gap between rural and urban areas for both frailty and functional limitation. Higher educational attainment and wealth in urban areas might be a factor responsible for lower frailty and functional limitations compared to rural areas. Physical activity was found to be negatively related with the urban-rural gap. The lower physical activity in urban areas might be led to decrease in the gap between urban and rural parts. For unexplained part, education and wealth quintile were positively significant for urban-rural gap. Working status and physical activity were negatively significant.

**Table 2:** Decomposition of frailty index and functional limitation by Place of residents among older persons

**Variables**	**Frailty (n=5347)**		**Functional limitation (n=5390)**	
	**Difference (95% CI)**	***P*** **-value**	**Difference (95% CI)**	***P*** **-value**
Rural	0.209 (0.205, 0.213)	0.000	45.155 (44.647, 45.669)	0.000
Urban	0.194 (0.187, 0.200)	0.000	42.410 (41.612, 43.213)	0.000
Difference	0.016 (0.008, 0.023)	0.000	2.745 (1.796, 3.695)	0.000
Explained	0.006 (0.002, 0.011)	0.009	1.053 (0.449, 1.657)	0.001
Unexplained	0.009 (0.002, 0.017)	0.017	1.693 (0.724, 2.661)	0.001
**Explained part**				
Age Group	0.000 (-0.002, 0.002)	0.925	0.011 (-0.189, 0.212)	0.912
Gender	-0.001 (-0.002, -0.001)	0.002	-0.239 (-0.379, -0.099)	0.001
Marital Status	0.000 (0.000, 0.000)	0.186	-0.020 (-0.053, 0.013)	0.243
Caste	0.000 (-0.001, 0.001)	0.389	-0.036 (-0.153, 0.081)	0.548
Religion	0.000 (0.000, 0.001)	0.100	0.022 (-0.021, 0.065)	0.311
Education	0.008 (0.006, 0.010)	0.000	1.095 (0.807, 1.383)	0.000
Wealth quintile	0.009 (0.007, 0.012)	0.000	1.183 (0.843, 1.524)	0.000
Currently working	-0.005 (-0.006, -0.003)	0.000	-0.536 (-0.709, -0.362)	0.000
Migration	0.000 (0.000, 0.001)	0.218	0.021 (-0.019, 0.060)	0.307
State	-0.001 (-0.001, 0.000)	0.078	0.016 (-0.059, 0.091)	0.674
Physical activity	-0.003 (-0.004, -0.002)	0.000	-0.376 (-0.515, -0.238)	0.000
Tobacco use	0.001 (0.000, 0.003)	0.037	0.128 (-0.033, 0.288)	0.120
Alcohol use	0.000 (0.000, 0.001)	0.718	-0.015 (-0.074, 0.044)	0.611
Community engagement	-0.002 (-0.003, -0.001)	0.000	-0.203 (-0.345, -0.062)	0.005
Personal engagement	0.000 (0.000, 0.000)	0.569	-0.009 (-0.030, 0.013)	0.427
Body mass index	Not applicable	Not applicable	0.011 (-0.206, 0.228)	0.922
**Unexplained part**				
Age Group	-0.000 (-0.002, 0.001)	0.925	0.011 (-0.189, 0.212)	0.912
Place of resident	-0.001 (-0.002, -0.001)	0.002	-0.239 (-0.379, -0.099)	0.001
Marital Status	0.000 (0.000, 0.000)	0.186	-0.02 (-0.053, 0.013)	0.243
Caste	0.000 (-0.001, 0.001)	0.389	-0.036 (-0.153, 0.081)	0.548
Religion	0.000 (0.000, 0.001)	0.100	0.022 (-0.021, 0.065)	0.311
Education	0.008 (0.006, 0.010)	0.000	1.095 (0.807, 1.383)	0.000
Wealth quintile	0.009 (0.007, 0.012)	0.000	1.183 (0.843, 1.524)	0.000
Currently working	-0.005 (-0.006, -0.003)	0.000	-0.536 (-0.709, -0.362)	0.000
Migration	0.000 (0.000, 0.001)	0.218	0.021 (-0.019, 0.060)	0.307
State	-0.001 (-0.001, 0.000)	0.078	0.016 (-0.059, 0.091)	0.674
Physical activity	-0.003 (-0.004, -0.002)	0.000	-0.376 (-0.515, -0.238)	0.000
Tobacco use	0.001 (0.000, 0.003)	0.037	0.128 (-0.033, 0.288)	0.120
Alcohol use	0.000 (0.000, 0.001)	0.718	-0.015 (-0.074, 0.044)	0.611
Community engagement	-0.002 (-0.003, -0.001)	0.000	-0.203 (-0.345, -0.062)	0.005
Personal engagement	-0.000 (0.000, 0.000)	0.569	-0.009 (-0.030, 0.013)	0.427
Body mass index	Not applicable	Not applicable	0.011 (-0.206, 0.228)	0.922

Similar results for gender gaps in disability rates were also observed (Table 3). Migration status was found to be negatively influencing the gender gap in disability. Higher migration among men may lead to increasing disability among them. Alcohol use positively influences the gender gap. Wealth quintile was also positively related to the gender gap. There was no significant difference in place of residents for disability rate, we still explored the analysis. Age, wealth index, and migration were found to be increasing the gap between rural and urban areas. While marital status found to be decreasing the gap between rural and urban areas, it suggests that unmarried/widowhood is more common in urban areas and turn, enhances disability in urban areas. For unexplained part, no variable was significant.

**Table 3:** Decomposition of Disability by gender and place of resident among older persons

**Variables**	**Decomposition by gender, n=9755**		**Dec omposition by place of resident, n=9755**	
	**Difference (95% CI)**	***P*** **value**	**Difference (95% CI)**	***P*** **value**
Female/Rural	0.189 (0.178, 0.199)	0.000	0.178 (0.168, 0.189)	0.000
Male/Urban	0.161 (0.151, 0.172)	0.000	0.173 (0.162, 0.184)	0.000
Difference	0.028 (0.013, 0.043)	0.000	0.005 (-0.010, 0.020)	0.479
Explained	0.013 (0.001, 0.025)	0.030	0.015 (0.009, 0.022)	0.000
Unexplained	0.014 (-0.004, 0.032)	0.117	-0.010 (-0.024, 0.004)	0.177
Explained part				
Age Group	0.001 (-0.001, 0.003)	0.296	0.004 (0.001, 0.006)	0.002
Marital Status	0.0148 (0.007, 0.023)	0.000	-0.002 (-0.003, -0.000)	0.006
Caste	0.000 (-0.000, 0.000)	0.929	-0.003 (-0.004, -0.000)	0.044
Religion	0.000 (-0.000, 0.000)	0.428	0.001 (0.000, 0.001)	0.026
Education	-0.000 (-0.004, 0.003)	0.793	0.000 (-0.003, 0.003)	0.934
Wealth quintile	0.001 (0.000, 0.002)	0.039	0.011 (0.004, 0.018)	0.004
Currently working	0.008 (0.003, 0.013)	0.003	-0.003 (-0.005, -0.001)	0.004
Migration	-0.001 (-0.013, -0.004)	0.000	0.003 (0.002, 0.005)	0.000
Tobacco use	-0.003 (-0.007, 0.000)	0.080	0.002 (-0.000, 0.004)	0.114
Alcohol use	0.005 (0.000, 0.010)	0.039	-0.000 (-0.001, 0.000)	0.145
living arrangement	-0.000 (-0.001, 0.001)	0.765	-0.000 (-0.001, 0.001)	0.762
State	-0.004 (-0.007, -0.002)	0.000	0.002 (-0.000, 0.005)	0.092
Unexplained part				
Age Group	0.028 (-0.032, 0.087)	0.358	-0.222 (-2.281, 1.837)	0.833
Marital Status	0.065 (-0.030, 0.161)	0.180	-0.063 (-0.705, 0.580)	0.849
Caste	0.061 (-0.052, 0.174)	0.293	0.444 (-3.597, 4.485)	0.829
Religion	0.009 (-0.032, 0.050)	0.668	0.114 (-0.920, 1.148)	0.829
Education	-0.003 (-0.017, 0.011)	0.680	0.007 (-0.078, 0.092)	0.875
Wealth quintile	0.026 (-0.036, 0.087)	0.416	-0.174 (-1.773, 1.425)	0.831
Currently working	0.008 (-0.006, 0.022)	0.262	-0.008 (-0.092, 0.077)	0.860
Migration	-0.017 (-0.038, 0.004)	0.103	-0.016 (-0.167, 0.135)	0.838
Tobacco use	-0.014 (-0.034, 0.006)	0.175	-0.070 (-0.715, 0.574)	0.831
Alcohol use	-0.000 (-0.004, 0.003)	0.825	0.030 (-0.243, 0.303)	0.831
Living arrangement	-0.008 (-0.128, 0.111)	0.890	0.487 (-3.955, 4.926)	0.830
State	-0.006 (-0.046, 0.034)	0.774	0.167 (-1.390, 1.724)	0.833

Regression results for frailty by gender and place of resident categories are given in Table 4. Education, wealth quintile, working status and physical activity were significantly influencing frailty among men. Marital status and religion were significant for females. Women belonging to Muslim and other religions were at lesser risk of frailty as compared to Hindu women. Age, gender and education were significant in both urban and rural areas. Increasing wealth was related to decreasing frailty index in urban areas, not found in rural areas. Current tobacco use was also positively associated with frailty in urban areas.

**Table 4:** Beta coefficient of Frailty among older person for gender and place of resident categories

**Variables**	**Male (n=2818)**	**Female (n=2529)**	**Rural (n=3920)**	**Urban (n=1427)**
Age group (yr)				
50-59	1.00	1.00	1.00	1.00
60-69	0.03 (0.02, 0.04)	0.03 (0.02, 0.04)	0.03 (0.02, 0.04)	0.04 (0.02, 0.05)
≥70	0.08 (0.07, 0.09)	0.09 (0.07, 0.10)	0.08 (0.07, 0.09)	0.10 (0.08, 0.12)
Gender				
Male	1.00	1.00	1.00	1.00
Female	Not applicable	Not applicable	-0.03 (-0.04, -0.02)	-0.04 (-0.06, -0.02)
Place of resident				
Rural	1.00	1.00	1.00	1.00
Urban	-0.01 (-0.02, 0.00)	-0.01 (-0.03, 0.00)		
Marital Status				
Married	1.00	1.00	1.00	1.00
Unmarried/ widowed/Separated	0.00 (-0.02, 0.01)	0.02 (0.01, 0.03)	0.01 (0.00, 0.02)	0.01 (-0.01, 0.02)
Caste				
SC/ST	1.00	1.00	1.00	1.00
Others	-0.01 (-0.02, 0.00)	0.00 (-0.01, 0.01)	0.00 (-0.01, 0.01)	0.00 (-0.02, 0.02)
Religion				
Hindu	1.00	1.00	1.00	1.00
Muslim	0.01 (-0.01, 0.02)	-0.03 (-0.05, -0.01)	-0.02 (-0.03, 0.00)	0.01 (-0.01, 0.03)
Others	0.00 (-0.03, 0.02)	-0.04 (-0.07, -0.01)	-0.02 (-0.05, 0.00)	0.00 (-0.03, 0.03)
Education				
Illiterate/ less than primary	1.00	1.00	1.00	1.00
Primary	-0.02 (-0.03, 0.00)	0.01 (-0.01, 0.02)	-0.01 (-0.02, 0.00)	-0.01 (-0.02, 0.01)
Secondary	-0.02 (-0.03, -0.01)	-0.02 (-0.04, 0.00)	-0.02 (-0.03, -0.01)	-0.02 (-0.04, 0.00)
Higher secondary and above	-0.04 (-0.05, -0.03)	-0.04 (-0.06, -0.02)	-0.04 (-0.05, -0.02)	-0.03 (-0.05, -0.01)
Wealth quintile				
Lowest	1.00	1.00	1.00	1.00
Lower	-0.02 (-0.03, 0.00)	-0.01 (-0.02, 0.01)	-0.01 (-0.02, 0.00)	0.01 (-0.03, 0.04)
Middle	-0.02 (-0.03, -0.01)	-0.01 (-0.02, 0.01)	-0.02 (-0.03, 0.00)	-0.01 (-0.04, 0.03)
Higher	-0.03 (-0.05, -0.02)	-0.02 (-0.04, 0.00)	-0.03 (-0.04, -0.02)	0.00 (-0.03, 0.03)
Highest	-0.05 (-0.06, -0.03)	-0.02 (-0.03, 0.00)	-0.03 (-0.05, -0.02)	-0.02 (-0.05, 0.01)
Currently working				
No	1.00	1.00	1.00	1.00
Yes	-0.05 (-0.06, -0.04)	-0.03 (-0.04, -0.02)	-0.05 (-0.05, -0.04)	-0.04 (-0.05, -0.02)
Migration				
No	1.00	1.00	1.00	1.00
Yes	0.00 (-0.01, 0.01)	0.00 (-0.02, 0.01)	0.00 (-0.01, 0.01)	-0.01 (-0.02, 0.00)
Physical activity				
Vigorous	1.00	1.00	1.00	1.00
Moderate	0.01 (0.00, 0.02)	-0.01 (-0.02, 0.00)	0.01 (0.00, 0.02)	-0.01 (-0.03, 0.00)
No activity	0.04 (0.03, 0.05)	0.03 (0.01, 0.04)	0.04 (0.03, 0.05)	0.01 (0.00, 0.03)
Tobacco use				
Never user	1.00	1.00	1.00	1.00
Past user	0.03 (0.01, 0.04)	0.08 (0.05, 0.12)	0.05 (0.04, 0.07)	0.03 (0.00, 0.06)
Current user	-0.01 (-0.02, 0.0)	0.02 (0.01, 0.03)	0.01 (0.00, 0.02)	0.01 (0.00, 0.02)
Alcohol use				
Never user	1.00	1.00	1.00	1.00
Past user	0.01 (-0.01, 0.02)	0.00 (-0.05, 0.05)	0.00 (-0.02, 0.01)	0.02 (0.00, 0.04)
Current user	-0.01 (-0.02, 0.00)	-0.02 (-0.06, 0.02)	-0.01 (-0.03, 0.00)	0.02 (-0.01, 0.04)

Regression results for functional limitations are given in Table 5. As expected, age was negatively associated with functional limitation in all gender and place of resident categories. Similar to frailty results, marital status and religion were found to be significant among females for functional health. Muslims and women belong to other religions were at lesser risk as compared to Hindu women. Age and education were significant in both urban and rural areas. Increasing wealth was related to decreasing functional limitations in urban areas, which was not true in the case of rural areas. Current alcohol use found to be improving functional health in urban, which was not significant for male, females and rural areas. Maintaining normal BMI was related with better functional health compared to lower BMI values. The regression result for disability from BKPAI data also shows similar results as frailty and functional health (Appendix 2). Education seems to have no relation to disability among older persons. Increasing wealth was significantly associated with disability in urban areas but not significant in rural areas. Currently working and migrated individuals have lesser disabilities compared to non-working and non-migrated older persons in both urban and rural areas.

**Table 5:** Beta coefficient of functional limitation among older person for gender and place of resident categories

**Variables**	**Male (n=2838)**	**Female (n=2552)**	**Rural (n=3968)**	**Urban (n=1422)**
Age group (yr)				
50-59	1.00	1.00	1.00	1.00
60-69	2.97 (1.78, 4.16)	2.92 (1.64, 4.20)	3.30 (2.28, 4.31)	3.89 (2.20, 5.57)
≥70	8.26 (6.77, 9.76)	10.38 (8.64, 12.13)	9.11(7.77, 10.44)	11.42 (9.25, 13.58)
Gender				
Female	1.00	1.00	1.00	1.00
Male	Not applicable	Not applicable	-4.45 (-5.86, -3.04)	-5.6 (-7.66, -3.53)
Place of resident				
Rural	1.00	1.00	1.00	1.00
Urban	-1.83 (-3.52, -0.13)	-2.05(-4.09, 0.00)	Not applicable	Not applicable
Marital Status				
Married	1.00	1.00	1.00	1.00
Unmarried/ widowed/Separated	-0.75 (-2.32, 0.81)	2.27 (1.01, 3.54)	1.27 (0.14, 2.39)	0.88 (-0.99, 2.74)
Caste				
SC/ST	1.00	1.00	1.00	1.00
Others	-0.81 (-2.17, 0.54)	0.56 (-0.98, 2.10)	-0.39 (-1.57, 0.79)	1.13 (-1.17, 3.43)
Religion				
Hindu	1.00	1.00	1.00	1.00
Muslim	1.40 (-0.43, 3.22)	-4.10 (-6.26, -1.94)	-1.82 (-3.6, -0.05)	0.73 (-1.91-3.38)
Others	1.06 (-2.36, 4.47)	-5.20 (-8.92, -1.48)	-2.84 (-6.21, 0.52)	0.33 (-3.73, 4.39)
Education				
No education/ less than primary	1.00	1.00	1.00	1.00
Primary	-2.40 (-3.84, -0.95)	0.21 (-1.68, 2.10)	-1.81 (-3.18. -0.44)	-1.78 (-3.96, 0.39)
Secondary	-2.77 (-4.37, -1.16)	-3.50 (-6.10, -0.91)	-2.39 (-4.11, -0.67)	-3.34 (-5.67, -1.01)
Higher secondary and above	-5.14 (-6.71, -3.56)	-5.11 (-7.93, -2.29)	-4.58 (-6.30, -2.86)	-4.45 (-6.74, -2.16)
Wealth quintile				
Lowest	1.00	1.00	1.00	1.00
Lower	-1.07 (-2.79, 0.66)	-1.35 (-3.26, 0.57)	-1.58 (-2.96, -0.19)	1.76 (-2.43, 5.95)
Middle	-1.53 (-3.29, 0.22)	-1.39 (-3.39, 0.61)	-1.52 (-2.99, -0.06)	-0.52 (-4.40, 3.35)
Higher	-3.38 (-5.16 -1.60)	-2.46 (-4.48, -0.44)	-3.41 (-4.93, -1.88)	-0.16 (-3.94, 3.62)
Highest	-5.09 (-7.01, -3.18)	-3.06 (-5.16, -0.96)	-4.03 (-5.67, -2.38)	-2.37 (-6.23, 1.48)
Currently working				
No	1.00	1.00	1.00	1.00
Yes	-6.07 (-7.25, -4.90)	-2.76 (-4.22, -1.31)	-5.11 (-6.17, -4.06)	-2.94 (-4.71, -1.17)
Migration				
No	1.00	1.00	1.00	1.00
Yes	-0.45 (-2.12, 1.22)	-0.22 (-1.68, 1.23)	-0.60 (-1.86, 0.66)	-0.69 (-2.40, 1.01)
Physical activity				
Vigorous	1.00	1.00	1.00	1.00
Moderate	1.19 (-0.04, 2.43)	-0.80 (-2.18, 0.58)	0.96 (-0.12, 2.04)	-2.26 (-4.03, -0.50)
No activity	5.20 (3.88, 6.52)	3.52 (1.88, 5.17)	5.81 (4.60, 7.03)	1.46 (-0.52, 3.44)
Tobacco use				
Never user	1.00	1.00	1.00	1.00
Past user	3.07 (1.01, 5.13)	11.36 (7.04, 15.69)	6.54 (4.35, 8.73)	3.02 (-0.40, 6.44)
Current user	-0.75 (-1.97, 0.48)	2.66 (1.34, 3.99)	1.20 (0.15, 2.25)	1.07 (-0.62, 2.76)
Alcohol use				
Never user	1.00	1.00	1.00	1.00
Past user	-0.16 (-1.61, 1.30)	0.46 (-5.77, 6.7)	-1.44 (-3.10, 0.22)	1.69 (-1.13, 4.51)
Current user	-1.56 (-3.19, 0.06)	-1.98 (-7.08, 3.12)	-2.33 (-4.09, -0.57)	1.84 (-1.66, 5.35)
Body mass index (kg.m^2^)				
<18	1.00	1.00	1.00	1.00
18-24	-1.29 (-2.41, -0.16)	-1.74 (-3.02, -0.45)	-1.86 (-2.82, -0.90)	0.56 (-1.35, 2.48)
25-29	-0.66 (-2.57, 1.26)	0.43 (-1.43. 2.28)	-0.55 (-2.25, 1.15)	2.07 (-0.25, 4.40)
≥30	-0.24 (-3.97, 3.49)	2.44 (-0.51, 5.40)	1.36 (-1.67, 4.39)	2.87 (-0.76, 6.51)

## Discussion

Most of the literature on gender differences in older populations focuses only on limitations in Activities of Daily Living (ADLs) and Instrumental Activities of Daily Living (IADLs) ^15, 24-27^. Not many studies have looked into gender differences in frailty, but abundant studies exist on functional limitation and disability. Like other studies conducted in other countries, we observed a female disadvantage ^27,28^. Our study is also one of the few studies explored many indicators of functional health. Our results are in line with the other studies suggesting around 50% of the inequality could be explained^28,29^. In our analysis education, wealth quintile, working status marital status and physical activity were significantly related with the gender gap in frailty, functional limitation. Women with higher educational attainment and living in the highest wealth quintile have less risk of functional limitation and disability which is in line with our findings^25,27,28,30^. Longitudinal studies in China and other countries have found a profound impact on education and wealth on frailty and functional health^30-32^. Similarly, maintaining weight and activity in later life is also found to be related to positive health outcomes^24,33^. Widowhood is another important factor influencing health of older women^28,34^. The unexplained part of the inequality suggests that there are factors that were either not assessed in the survey or were not included in the present analysis are responsible for the differences. This is a strength rather than a limitation. Breaking down the inequality in this way provides a platform for further research to understand relevant factors for policies catering gender inequalities.

The results point to greater inequalities in disability between men and women and urban and rural residents^2,3^. The education, wealth and infrastructure inequality that exists between urban and rural India is also important in influencing the health of older persons^35,36^. Our decomposition analysis showed that these inequalities are mainly attributed to social factors such as marital status, employment and education. These factors are also associated with restrictions on women’s mobility and social connectivity^28,37^. Counting based measures of disability such as functional limitation and frailty combined with self-reported disability using multiple data sets confirm the female disadvantage in India. Except for the biological factors, discrimination of women in social and economic aspects are also responsible for the gender differences in India^28^.

The poorer functional health among older women, compared with men in India can be largely explained by the gender differentials in socioeconomic status and consequent empowerment (such as less control of their mobility and financial independence) ^26,28^. The gender discrimination is reflected through many social and economic inequalities. These disparities must be understood targeted by public health strategies to improve health among older persons ^38^. Urban and rural divide in health status also provides an interesting picture. The results of our study might suggest that remaining active and engaged can help in improving physical health at age, supporting continuity theory ^39^. The health disparity in gender and place of residence, which already present may represent a challenge rise as the demographic transition speed up in India ^9^. Efforts to improve gender disadvantages in various life stages might be an important attempt in improving health among females. On similar line, the social welfare benefits including age pensions and health insurance are not yet universal. Our results indicated that older women and rural residents are more likely to be excluded in accessing affordable health care as compared to their counterparts.

This analysis is based on cross-sectional data and cannot address causality. This suggests a need for further research to identify other factors that contribute to these inequalities. These issues should be considered when planning future surveys seeking to examine social and spatial diversities in physical and functional health among older persons.

## Conclusion

The higher female disadvantages in frailty and functional health can be explained by education, working status, physical activity and migration status. The factors responsible for urban-rural differences were education, wealth quintile, working status and community engagement. These are also the factors that may represent the life course gender discrimination. Efforts to improve gender disadvantages in earlier life stages might get reflected in better health for females in older age. As the demographic and economic developments are speeding up in India, there would be a focus on improving the social and economic mobility of women in India.

## Acknowledgements

None.

## Conflict of interest

The authors have no conflict of interest to declare for this study.

## Funding

None.

## Highlights

- Education and Wealth quintiles are related to gender disparities.
- Low physical activity and non-working status are also important factors.
- Efforts to improve gender disadvantages in various life stages are necessary.
- Economic development must cater to the health needs of women and rural dwellers.
